# Parliament and the Profession

**Published:** 1915-12-11

**Authors:** 


### PARLIAMENT AND THE PROFESSION.

### The Week in the House of Commons.

Questions of medical interest asked in the House of Commons since The Hospital of last week went to press have dealt with a variety of topics. In point of number, though not of importance, matters arising out of the administration of the Insurance Act have held the first place; but as most of the questions thereon drew replies of a not altogether definite character, it is unnecessary to refer to them here. There seems to be an evasive tendency—possibly unintentional on the part of Ministers—in the replies to some of these questions; and the same applies to inquiries concerning the treatment of tuberculous soldiers. This is unfortunate. We should like to see questions answered more frankly and with more directness.

### Asylum Attendants of Military Age.

Having in mind, no doubt, the desirability of releasing as many men as possible for Army service, Sir John Lonsdale asked the Chief Secretary for Ireland if asylum committees had power to require that candidates for positions as attendants must not be of military age or fit for Army service. In his reply Mr. Birrell intimated that if the regulations did not give the committees a sufficiently wide discretion, any alteration proposed in the direction suggested would, of course, be sympathetically considered.

### Ex-Clerks as Hospital "Charwomen."

Mr. Jowett has drawn the attention of the House of Commons to the fact that ex-clerks who have joined the R.A.M.C., and whose vacated posts have been filled by women, are being employed to a considerable extent instead of charwomen in British hospitals. Mr. Tennant's explanation of this is that the men in question are so employed in order that they may have experience of the work they will be required to do when they are employed in hospitals abroad. It is regarded as a necessary part of their training.

### Lord Derby and Third-Year Medical Students.

On November 30 Mr. Snowden asked the Under-Secretary for War if he would exempt from pressure medical students who are approaching the end of their third year's course. Mr. Tennant's reply was to the effect that students who at or before the close of the present winter session will be qualified for entry to one of the examinations for the third-year students in medicine and duly enter for the examination for which they are now studying will not be attested until after its conclusion, and, if they are successful, will be included in the class of fourth-year men under Lord Derby's scheme. Mr. Tennant added that he could not give instructions that first and second year students who desired to continue their training should be allowed to do so any more than he could undertake "to cancel the decision that candidates for holy orders are to be regarded as eligible to be canvassed and enlisted or to receive commissions." Why drag in the parsons?

### The Position of Tuberculous Soldiers.

The problem of the after-treatment of soldiers discharged from the Army suffering from tuberculosis is likely to become a serious one in the near future. In spite of the reassuring statements made by Ministers in the House of Commons, the uncomfortable feeling is steadily growing that the arrangements that have been made for the treatment of such men are inadequate. It was alleged by Mr. R. McNeill on December 2 that in some cases the discharge from the Army takes effect when the men are in a dying condition, and that the War Office refuses even to provide burial in such cases. If this is a fact, it reflects behaviour on the part of the authorities that is heartless, cruel, and disgraceful. There is something about the tone of the replies of Ministers to the many questions relating to tuberculous soldiers that is unsatisfactory. The public is more concerned to know definitely what provision has been made for their treatment than to be told that in the great majority of cases the disease is not due to military service.

### Badges for Admiralty Dentists.

On December 2 the First Lord of the Admiralty was asked by Mr. R. McNeill what was the reason for the discrimination between dental surgeons in the service of the Admiralty, whereby those afloat are given commissions and those ashore are not given commissions, although the qualifications and duties of the two classes are identical. Mr. McNeill also desired to know whether under Lord Derby's scheme of recruiting for the Army dental surgeons employed ashore by the Admiralty are held to be indispensable, and are not eligible for enlistment. Dr. Macnamara, who replied, pointed out that there were obvious differences in the conditions of service of dentists at sea and those serving ashore, and the claims of the latter were fully considered in arriving at the decision not to grant them commissions. The services of dental surgeons employed on shore are indispensable, and in view of this they have been granted the Admiralty war service badge, and will in due course be given a special certificate stating that their services cannot be spared, and that it is their duty to remain at their present posts.

### Other Matters of Interest.

Vaccination Essential.—Persons offering themselves for enlistment under Lord Derby's scheme are in all cases required to be vaccinated. Certain men who have offered themselves have stated that they were unwilling to be vaccinated, and have, in consequence, been told that they could not be attested. This was the gist of a statement made by Mr. Tennant on December 2.

Scotch Panel Chemists.—It is evident that there has been some bungling in the negotiations between the Insurance Act authorities and Scottish panel chemists with regard to the enforcement of the new drug tariff. One of the few things that seem to emerge with any clearness from the various statements made in the House is that the chemists have refused to accept the new terms, and that unless the Commissioners meet the wishes of the chemists Scotland will shortly be without a Pharmaceutical Insurance Service.

National Insurance.—On the motion for the adjournment of the House on December 1 Mr. Currie called attention to the operation of the Insurance Act. He stated that the condition of sanatorium benefit was really one of partial paralysis, and that there was a widely prevalent feeling that working men who paid the premiums were not getting enough back. Mr. C. Roberts, replying for the Government, denied that the sanatorium benefit had broken down. On the contrary, the progress had been great, though something remained to be done. It was true, he said, that the war had had some effect on the finances of the Act, but there was no foundation for the accentuated apprehension displayed.

